# No association between XMRV or related gammaretroviruses in Australian prostate cancer patients

**DOI:** 10.1186/1743-422X-10-20

**Published:** 2013-01-10

**Authors:** Simin D Rezaei, Anna C Hearps, John Mills, John Pedersen, Gilda Tachedjian

**Affiliations:** 1 Retroviral Biology and Antivirals Laboratory, Centre for Virology, Burnet Institute, 85 Commercial Road, Melbourne, Victoria, 3004, Australia; 2 Department of Microbiology, Monash University, Clayton, Victoria, 3168, Australia; 3 Department of Medicine, Monash University, Melbourne, Victoria, 3004, Australia; 4 TissuPath, Specialist Pathology, Mount Waverley, Victoria, 3149, Australia

## Abstract

**Background:**

Xenotropic murine leukemia virus-related virus (XMRV) is a gammaretrovirus reported to be associated with prostate cancer (PC) and chronic fatigue syndrome (CFS). While the association of XMRV with CFS and PC has recently been discredited, no studies have been performed in Australian patients to investigate the association between PC and XMRV or related murine leukemia virus (MLV) in matched PC and normal tissue.

**Methods:**

Genomic DNA (gDNA) was purified from matched normal and cancer formalin-fixed paraffin-embedded (FFPE) prostate tissue from 35 Australian PC patients with Gleason scores ranging from 7 – 10. The presence of the ribonuclease L (RNase L) polymorphism R462Q was determined by allele specific PCR. Samples were screened for XMRV and related murine leukemia virus (MLV) variants by qPCR. Contaminating mouse DNA was detected using qPCR targeting mouse intracisternal A particle long terminal repeat DNA.

**Results:**

gDNA was successfully purified from 94% (66/70) of normal and cancer FFPE prostate tissues. RNase L typing revealed 8% were homozygous (QQ), 60% were heterozygous (RQ) and 32% were wild-type (RR) for the RNase L mutation. None of the 66 samples tested were positive for XMRV or related MLV sequences using broad MLV or XMRV specific primers with detection sensitivities of 1 viral copy of MLV/XMRV and XMRV DNA, respectively.

**Conclusions:**

Using highly sensitive qPCR we found no evidence of XMRV or related gammaretroviruses in prostate tissues from 35 Australian PC patients. Our findings are consistent with other studies demonstrating that XMRV is a laboratory contaminant that has no role in the aetiology of PC.

## Introduction

Prostate cancer (PC) is one of the most commonly diagnosed cancers in men resulting in approximately 3,300 deaths in Australia per year. While the aetiology of PC remains poorly understood, infection and associated inflammation are risk factors in PC development [1]. The rationale for a recent search for a viral origin for PC was based on the observation that a reduced activity variant of the antiviral *RNASEL* gene (R462Q) was associated with familial PC [2]. A gammaretrovirus named xenotropic murine leukemia virus-related virus (XMRV) was identified in cDNA samples from seven of 11 R462Q homozygous (QQ) cases using a novel DNA microarray (Virochip) containing oligonucleotides comprising conserved sequences from known viral genomes [2]. However, the association of XMRV with the QQ RNASEL variant was observed in some [3] but not all studies [4,5]. XMRV was reportedly linked with higher-grade PC cancers suggesting that its presence may be a useful biomarker for severe disease [4]. However, there was discordance with regard to the cellular location of XMRV in the prostate where positive signals by fluorescence *in situ* hybridization (FISH) and immunohistochemistry assays were observed in either malignant epithelium [4] or stromal cells [2,3].

Gammaretroviruses comprise a group within the larger retrovirus family that reverse-transcribe viral RNA to a cDNA during replication that is inserted into the host cell chromosome. As the name indicates, XMRV is highly related to murine leukemia virus (MLV) sharing 96% sequence identity [2]. In addition to PC, XMRV was also associated with chronic fatigue syndrome (CFS) [6], which was remarkable since no other gammaretroviruses have been described that infect humans. However, XMRV related xenotropic-MLV (X-MLV) are known to infect other species including mice, koalas, cats and non-human primates and cause leukemias, lymphomas, neurological diseases and immunodeficiencies in these species suggesting a plausible role for XMRV in PC [4].

In addition to the original report by Urisman and colleagues (2006) several other groups demonstrated an association of XMRV with PC using nucleic acid detection and immune based assays [3-5]. However, many other studies either failed or were only able to detect XMRV in a minority of PC tissue samples [7-19]. While the reason for the reported disparity of XMRV prevalence in PC was unclear, it was initially attributed to differences in geography or assay sensitivity. However, subsequent studies demonstrated that positive signals in sensitive PCR assays could be ascribed to either mouse DNA contamination or contamination with XMRV DNA from a commonly used PC cell line (22Rv1), which harbours 10–20 copies of XMRV [20-26]. The discovery that XMRV was generated by recombination of two mouse endogenous retroviruses following passage of a PC xenograft in nude mouse demonstrated that XMRV was generated in the laboratory [27].

Due to public health consequences of potential infection with XMRV or MLVs in humans, we considered it is important to refute or confirm their association with PC in an Australian context. This study describes the first evaluation in Australian PC patients for the presence of XMRV and related gammaretroviruses in matched normal and PC tissue and the elucidation of the RNASEL genotype in Australian PC patients. In addition, patient samples were tested for the presence of mouse DNA contamination using a sensitive quantitative PCR (qPCR) assay that detects mouse intracisternal A-type particle (IAP) sequences. We failed to detect XMRV or related MLVs in Australian PC patients confirming that these gammaretroviruses are highly unlikely to be involved in PC.

## Results

### Patient characteristics and RNase L genotype

The patient population comprised individuals with higher-grade PC as determined by Gleason scores (median 7, range 7 – 10). Consistent with PC predominately presenting in older men, the median age of patients was 64 (range 49 – 78). The RNase L genotype was determined using purified DNA recovered from 70 formalin-fixed paraffin-embedded (FFPE) normal and cancer tissue from each of 35 patients. The RNase L genotype of both samples from each patient was 100% concordant and 8% of the patients were homozygous (QQ), 60% were heterozygous (RQ) and 32% were wild-type (RR).

### Human VAMP2 qPCR confirms integrity of DNA purified from FFPE samples

DNA derived from FFPE samples is expected to be fragmented due to the fixation process and may contain substances that are inhibitory for PCR amplification. Therefore, to evaluate the suitability of genomic DNA (gDNA) derived from FFPE tissue for qPCR, we determined whether we could amplify a region of the single copy human vesicle-associated membrane protein 2 (HuVAMP2) gene. The size of the amplimer (78 bp) was designed to be similar to the expected amplimer sizes for qPCR detection of XMRV and MLV-related viruses (Table 1). The linear range and sensitivity of human VAMP2 qPCR was 1 to 10^7^ copies per reaction (Figure 2A). All samples tested gave HuVAMP2 values within the acceptable range and were thus deemed suitable for further qPCR analysis. Mean HuVAMP2 copy numbers from gDNA purified from normal and cancer tissue were similar (*P* = 0.7, n = 33, Mann–Whitney *U* Test).

**Table 1:** PCR primers and probes

**Target**	**Amplicon Size**	**Method**	**Primers and TaqMan Probe (5’-3’)**	
HuVAMP2^1^	78 bp	qPCR	HuVAMP2-F	CAGCATCTCTCCTACCCTTTCAC
			HuVAMP2-R	CCCCACACTTCTGGTTTTCTG
			Huvamp2-Probe	6FAM-AGCAGGGATATCTAAGC-MGBNFQ^2^
BMLV^3^	76 bp	qPCR	BMLV-F	GCCTGTCCAGGATCTGAGAG
			BMLV-R	GAGGTTGTAAGGGTTGGGCA
			BMLV- Probe	5FAM-AAGTCAACAAGCGGGTGGAAGABHQ^4^
XMRVIN^5^	70 bp	qPCR	XMRVIN-F	CGAGAGGCAGCCATGAAGG
			XMRVIN-R	GCGTATACGGGGTTGAGTCC
			XMRVIN-Probe	6FAM-AGTTCTAGAAACCTCTACACTC-MGB
IAP^6^	71 bp	qPCR	MIAP-F	GCCGCGCCCACATT
			MIAP-R	CGCAGATTATTTGTTTACCACTTAGAA
			MIAP-Probe	6FAM-CCGTTACAAGATGGTGCTGA-MGBNFQ
RNASEL	137 bp	PCR	462R-F	GTGGAAAATGAGGAAGATGAATTTGCCAG
			462Q-F	GTGGAAAATGAGGAAGATGAATTTGCCAA
			462-R	ATTGGGGACTCACCTATTAAGATGTTTTG

^1^Human vesicle associated membrane protein 2.

^2^Minor group binding (MGB) non-fluorescent quencher.

^3^Primers can detect both XMRV and MLV sequences.

^4^Black hole quencher.

^5^Primers/probe specifically detect conserved integrase sequences.

^6^Mouse intracisternal-A particl.

**Figure 1:**
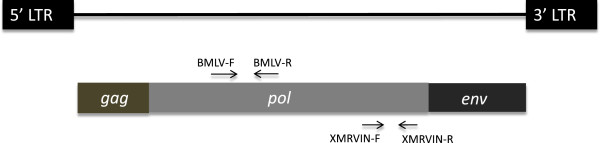
Genomic organization of XMRV and MLV showing the targets of broad MLV forward (BMLV-F) and reverse (BMLV-R) primers and the XMRV specific forward (XMRVIN-F) and reverse (XMRVIN-R) primers.

### XMRV or related MLV sequences were not detected in gDNA from paired normal and cancer tissue

**Figure 2:**
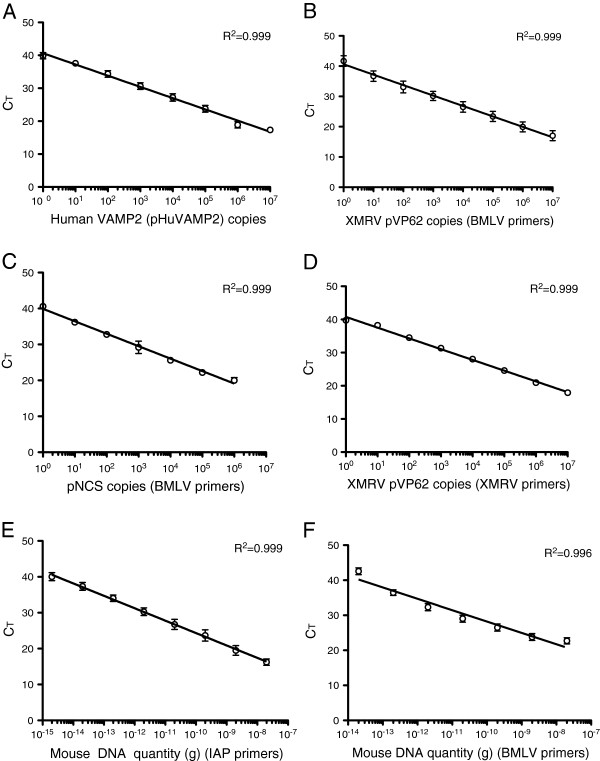
**Linear regression analysis demonstrating the linear range and sensitivity of qPCR assays used to detect human VAMP2 in pHuVAMP2 using HuVAMP2 primer/probes (A), XMRV in VP62 using BMLV primers/probe (B), MoMLV in pNCS using BMLV primers/probe (C), XMRV in VP62 using XMRV-IN specific primers/probe (D), Balb/c DNA using IAP primers/probe (E) and Balb/c DNA using the BMLV primers/probe (F).** Plasmid targets at the indicated copy numbers or Balb/c DNA at the amounts shown were subjected to qPCR in the presence of 1 μg of tRNA as the carrier nucleic acid and the logarithm of these values were plotted against the threshold cycle (CT) value. All data points were derived from triplicate wells and the error bars denote the standard deviation. Data shown are representative of three independent assays except for detection of XMRV in VP62 with XMRVIN specific primers (**D**) and Balb/c DNA detection with the BMLV primers/probe (**F**), which were performed once. R^2^ denotes the Pearson correlation coefficient.

To detect XMRV and related MLV sequences in a single qPCR reaction we used broad MLV (BMLV) primers targeting the conserved reverse transcriptase regions of XMRV and Moloney murine leukemia virus (MoMLV)(Figure 1). The linear range and sensitivity of the BMLV primers/probe used for amplification of XMRV and MoMLV was determined to be 1 to 10^7^ and 1 to 10^6^ copies per reaction, respectively (Figures 2B and 2C). We also evaluated the linear detection range and sensitivity of XMRV specific primers targeting the integrase region of XMRV (Figure 1) [28] and determined it to be from 1 to 10^7^ copies of XMRV per reaction (Figure 2D). Taken together these data demonstrate that the qPCR assays reproducibly detected as little as 1 copy of XMRV and MoMLV in a total of 1 μg of carrier nucleic acid.

We performed qPCR using BMLV primers on gDNA purified from 33 normal prostate and 33 PC FFPE tissue. DNA was subjected to three independent assays except for 10/33 cancer and 16/33 normal tissues where only one qPCR assay was performed due to limited amounts of gDNA. All patient samples were negative using the BMLV primers (Table 2). We also subjected DNA purified from 19 normal and 27 cancer samples to qPCR using the XMRV specific primers and again found that all samples were negative (Table 2). For both the BMLV and XMRV specific qPCR assays, no amplification was observed in any of the 12 negative control wells while all positive control wells (containing 22Rv1 DNA) were consistently positive. These data demonstrate that XMRV or related MLV were not detected in prostate tissue tested in this study.

**Table 2:** Amplification of XMRV, MLV sequences and mouse IAPs in cancer and normal prostate tissue by qPCR

	**Target**		
**Patient Tissue**	**BMLV**^**1**^	**XMRV**	**Mouse IAP**
Normal	0/33^2^	0/19^3^	5/33^2^
Cancer	0/33^2^	0/27^3^	10/33^2^

^1^Detection of both XMRV and MLV sequences.

^2^qPCR performed in 3 independent assays, except for 10/33 cancer and 16/33 normal prostate tissue, where qPCR was performed once.

^3^qPCR performed once.

### Evidence of mouse DNA contamination in PC samples

To determine whether patient samples were contaminated with mouse DNA we performed qPCR with primers that detect the mouse IAP retrotransposon [28,29]. The mouse genome contains approximately 1000 IAP copies [30], thus detection of IAPs is a highly sensitive method for assessing mouse DNA contamination. Evaluation of the linear range and sensitivity of the qPCR IAP assay using purified Balb/c mouse gDNA showed that the assay was highly sensitive, detecting 2 fg to 20 ng of mouse gDNA (Figure 2E). The BMLV qPCR assay detected from 20 fg to 20 ng of endogenous MLV present in Balb/c gDNA (Figure 2F) indicating that this assay was 10-fold less sensitive in detecting Balb/c gDNA than the qPCR IAP assay.

We assessed mouse DNA contamination in gDNA from FFPE patient samples in qPCR IAP assays and found that 5/33 and 10/33 of normal and cancer tissues, respectively were positive for mouse IAP (Table 2). The level of mouse DNA contamination varied from 2 – 20 fg (data not shown). These data show that 23% (15/66) of patient samples were positive for low-level mouse DNA contamination.

## Discussion

Using highly sensitive qPCR neither XMRV nor related MLV were detected in any of the prostate tissue samples in our Australian cohort. The BMLV qPCR assay used in this study reproducibly detected as little as one copy of XMRV or MoMLV DNA in a total of one μg of carrier nucleic acid. While several studies have reportedly used primers that simultaneously detect XMRV and related MLV in PC samples [10,12,13,18,31], only one of these studies reported a similar sensitivity of XMRV detection [18] compared to this study. Also, in contrast to our study, validation of the sensitivity of primers for detecting other MLV such as MoMLV was not reported. The reverse transcriptase target of the BMLV qPCR assay is highly conserved demonstrating 97-100% sequence identity with preXMRV-1, preXMRV-2, Friend MLV, Friend spleen focus-forming virus and Rauscher MLV indicating that our assay could potentially detect other MLV gammaretroviruses apart from XMRV and MoMLV. Thus the absence of XMRV or related MLV in PC tissue samples in this study is consistent with previous findings negating a role for these viruses in PC.

We obtained paired normal and cancer tissue from the same patient to determine whether there was a difference in prevalence of XMRV or related MLV in these tissues to provide clues to their role in PC pathogenesis. This is in contrast to previous studies that have used 10 μm patient tissue slices that are likely to be a mixture of both cancer and normal tissue [4]. Our strategy also enabled us to maximise the amount of cancer tissue recovered to increase the probability of detecting XMRV or related MLV in samples. We were able to selectively obtain cancer and normal tissue by using histological staining of tissue slices to identify appropriate regions in the FFPE prostate tissue for obtaining the punch biopsies. In addition, we developed methods to purify DNA from FFPE tissue core biopsies suitable for downstream qPCR analysis that may be valuable for other studies requiring recovery of DNA for nucleic acid detection.

Previous studies have raised serious doubts regarding the role of XMRV in PC and CFS. The first studies to cast doubt on this association demonstrated that PCR reagents and nucleic acid purification columns were contaminated with mouse DNA that harbours MLVs detected by highly sensitive PCR [20-24]. In addition, the specificity of putative XMRV-specific primers detecting a 24-nucleotide *gag*-leader deletion was challenged with the finding that these primers were able to amplify endogenous MLV sequences present in the gDNA of 12 different mouse strains [25]. These studies advocated the inclusion of sensitive counter assays to detect mouse DNA contamination to verify XMRV positive samples [13,28]. However, the remarkable nucleotide sequence identity of XMRV from diverse patient samples remained a conundrum since retroviral polymerases are error prone and therefore XMRV detected from distinct sources would be expected to show greater sequence diversity. This finding pointed to yet another source of patient sample contamination. Phylogenetic analysis of XMRV sequences from unlinked patients and a commonly used PC cell line (22Rv1) showed that these sequences formed a monophyletic clade and that the cell line-derived sequences were ancestral to the patient-derived sequences (posterior probability >0.99). These findings led to the conclusion that XMRV contamination originated from the 22Rv1 cell line [25,26]. Furthermore, the possibility that XMRV in 22Rv1 cells originated from a *bone fide* human infection was debunked by Paprotka and colleagues who showed that XMRV was a laboratory virus generated by a rare recombination event between two mouse endogenous retroviruses during passage of the CWR22 PC xenograft in nude mice from which the 22Rv1 cell line was derived [27].

We used a previously published IAP qPCR assay [28], to detect mouse DNA contamination in samples, which in our hands achieved a similar detection sensitivity of 2 fg of mouse gDNA. While we did not detect XMRV or related MLV by qPCR in any of the patient samples, 15% of normal and 30% of cancer tissues were positive for mouse IAP, which was reproducibility observed in 2/3 independent assays in three samples and 3/3 assays in 12 samples. The level of contamination was low and in the range of 2 – 20 fg/μg of patient DNA, which likely explains the failure to detect mouse DNA in these samples using the BMLV qPCR, which has a limit of detection of 20 fg/μg gDNA.

While mouse DNA contamination was detected in seven cancer samples in the IAP qPCR assay, no signal was observed in normal samples from the corresponding patients. Due to the limited amount of gDNA we were unable to test normal prostate tissue from one of these seven patients. In contrast, both normal and cancer samples from three patients were positive for mouse DNA contamination. The greater number of mouse DNA positive samples in cancer compared to normal tissue is unlikely due to increased processing of the former samples because the samples were all handled a similar number of times. In addition, it is unlikely that PCR reagents used in this study were contaminated with mouse DNA as none of the no template controls (12 wells per plate) or DU145 and LNCaP gDNA negative controls (3 wells of each per plate) were positive for XMRV or related MLV in our qPCR assays. Furthermore, we avoided using a Taq polymerase that relies on a monoclonal antibody to achieve a “hot start” as this has previously been implicated as a source of mouse DNA contamination [22,23]. The DU145 and LNCaP gDNA controls were also included to determine whether there was mouse DNA contamination from DNA extraction columns [24]. While we did not observe any positive signals in these controls, the sporadic nature of this contamination makes it difficult to exclude this possibility. In addition, procedures were implemented to prevent cross contamination of samples during tissue biopsy collection at TissuPath; however, the presence of mouse DNA during the original processing of the samples cannot be excluded. Therefore, the contamination observed is likely to be random and possibly due to the DNA extraction columns and/or contamination during the original fixation and paraffin embedding of the prostate tissue, microtome sectioning of samples or preparation of the punch biopsies for this study.

One potential limitation of our study is that we used naked plasmid DNA for determining the analytical range of the qPCR assays used in this study while the samples were formalin-fixed DNA from tissue. This may have led to possible overestimation of the sensitivity of the assays. Regardless, our findings are consistent with previous studies demonstrating no association between XMRV and related gammaretroviruses in prostate cancer patients undertaken in regions geographically distinct from Australia.

The original premise for determining the *RNASEL* genotype of patients in our cohort was to establish if there was an association with the homozygous (QQ) RNase L variant and XMRV or related MLVs. The RNase L enzyme is an interferon-induced ribonuclease, which has antiviral activity and can also induce apoptosis [32,33]. Men that are heterozygous or homozygous for the mutant form of the allele have 50% and greater than 2-fold increased risk, respectively of PC than non-carriers [34]. Given the role of RNase L in antiviral defense, it has been proposed that a viral infection may contribute to PC [2]. Since none of our samples were positive for XMRV or related MLVs, an association between *RNASEL* mutation and infection with these viruses could not be investigated. Regardless, we successfully determined the *RNASEL* genotype for all DNA samples purified from FFPE cancer and normal prostate tissue using a previously published allele specific PCR assay [34]. The genotypes determined for cancer and normal prostate tissue from the same patient, which were performed blinded, were 100% concordant. To our knowledge this is the first time that the *RNASEL* genotype of Australian PC patients has been determined. The overall allele distribution in our small cohort appears to be similar to non-hereditary PC cases observed in the USA where the heterozygous (RQ) allele has the greatest prevalence (~47%) and the homozygous (QQ) allele the lowest prevalence (9.9 – 15.2%) [4,35]. Further studies with a larger cohort would be of interest to determine whether the *RNASEL* allele distribution in Australian PC patients are distinct to normal men in the same geographical region, if there are racial differences, and whether there is an association of this allele with disease severity.

## Conclusions

The Blood XMRV Scientific Working Group’s findings, along with the discovery that XMRV is a virus generated by a rare recombination event in the laboratory has provided irrefutable evidence that XMRV or related MLV are not associated with CFS [27,36,37]. These reports have lead to the retraction of the original study describing the association of XMRV with CFS [6,38]. In addition, a separate study has confirmed that XMRV or closely related viruses were not present in the primary tissues from which the XMRV-infected cell line 22Rv1 was derived [31]. Furthermore, strong evidence for XMRV infection of human cells in the prostate as demonstrated by XMRV DNA joined to human DNA sequences has been found to be due to DNA contamination from XMRV infected DU145 PC cells used in the same laboratory [26,39]. The absence of XMRV and related MLV in Australian PC patients using a highly sensitive pPCR assay is consistent with previous reports concluding that XMRV and related MLV positive signals in patient samples are due to contamination and that there is no causal link between these gammaretroviruses and PC.

Added in proof: Following submission of our study a report by Lee and colleagues [40] demonstrated that archival RNA from prostate cancer samples used in the first study reporting an association between XMRV and PC [2] was contaminated with XMRV originating from a XMRV-infected cell line which led to the retraction of the original report in PLoS Pathogens. In addition, a multicenter-blinded analysis headed by Ian Lipkin which analyzed peripheral blood from well-characterized and geographically diverse populations of CFS and myalgic encephalomyelitis (MS) patients demonstrated no association with either XMRV or polytropic MLV [41].

## Methods

### Study population and specimens

Prostate samples used in this study were archival FFPE tissue obtained from patients in the greater Melbourne area who had radical prostatectomies performed with tissues submitted to TissuPath Specialist Pathology (Mount Waverley, Victoria, Australia) for diagnostic pathology between 2007 and 2011. For this study, the samples were prepared by TissuPath scientists from normal and cancer affected regions of the prostate, guided by the associated haematoxylin and eosin (H&E) stained tissue sections which had been evaluated by light microscopy. Samples were received as 2–3 FFPE punch biopsies of 2 mm × 2 mm (diameter × depth). Specimens, coded to mask whether they were normal or cancer tissue and to maintain patient confidentiality, were provided to investigators at the Burnet Institute. Samples were unblinded following completion of the assays at which time the age and Gleason scores for each patient was provided. The study was approved by the Alfred Health Human Ethics Committee (Project Number 32/11).

### Cell lines

The human prostate carcinoma cell line 22Rv1 [42] and the human embryonic lung fibroblast cell line MRC-5 [43], were obtained from the American Type Culture Collection (ATCC, Manassas, VA, USA). Human prostate carcinoma cell lines, DU145 [44] and LNCaP [45] were provided by Renee Taylor and Gail Risbridger (Monash University, Clayton, Australia). Human peripheral blood mononuclear cells (PBMCs) were isolated from donor buffy coat packs (from donors screened for the absence of blood pathogens) obtained from the Australian Red Cross (Melbourne, Australia) and purified by Ficoll-Paque™ PLUS centrifugation according to manufacturer’s instructions (GE Healthcare, Uppsala, Sweden).

### Plasmids

The plasmid, pVP62, encodes the full length molecular clone of XMRV inserted in the mammalian expression vector pcDNA3.1(−) and was obtained from the NIH AIDS Research & Reference Reagent Program [2]. pNCS, a gift from Stephen Goff (Columbia University, New York, USA) encodes the full length molecular clone of MoMLV and is a derivative of pNCA carrying an SV40 origin of replication in the plasmid backbone [46]. pHuVAMP2, harbouring the human vesicle-associated membrane protein 2 (VAMP2) gene, was generated by PCR amplification from gDNA purified from DU145 cells using HuVAMP2-F and HuVAMP2-R primers (Table 1). The 78 bp amplicon was cloned into the TOPO TA vector according to manufacturer’s instructions (Invitrogen, Carlsbad, CA, USA) and the identity of the clone verified by nucleotide sequencing.

### Laboratory techniques to prevent sample contamination

To avoid cross contamination each tissue sample was obtained using a separate sterile 2 mm punch biopsy. To minimize the exposure of patient samples to potential sources of mouse, XMRV and MLV DNA, standard laboratory procedures for sterile DNA extraction were practiced when handling and processing specimens for purification of gDNA and PCR. These measures included the use of sterile UV irradiated microcentrifuge tubes, filter-barrier pipette tips and dedicated micropipettes. All tissue processing was performed in a biosafety class II cabinet and equipment was exposed to UV light for 15–20 min prior to use.

### Genomic DNA purification from FFPE prostate tissue and cell lines

gDNA was purified from FFPE patient tissue using the QIAamp® DNA FFPE Tissue Kit and the QIAGEN Deparafinisation solution (DPS, QIAGEN, Hilden, Germany). Since the QIAamp kit recommends extracting gDNA from tissue sections of 10 μm in thickness, we introduced modifications to optimise DNA recovery from the thicker core biopsies used in this study. These modifications included trimming excess paraffin from the core biopsies, dissecting the tissues into a maximum of five pieces prior to the addition of DPS, and an overnight incubation of tissues with proteinase K. gDNA was extracted from a total of 70 prostate tissue punch biopsies. gDNA was purified from DU145, LNCaP, MRC-5, 22Rv1 cells and PBMCs using the DNeasy Blood and Tissue Kit (QIAGEN) according to manufacturer’s instructions.

### Amplification refractory mutation system (ARMS) to detect RNase L R462Q polymorphisms

The RNase L R462Q polymorphism in patient samples was determined using the ARMS assay as previously published [34]. ARMS is an allele specific PCR assay that uses two forward primers with different 3’-termini to specifically detect either the wild-type R462 (462R-F) or the mutant R462Q (462Q-F) allele, while the reverse primer (462-R) detects both alleles (Table 1). Each assay included control DNA from DU145, LNCaP and 22Rv1 PC cell lines that have the RQ, RR and QQ RNase L genotypes, respectively and 20 ng of gDNA from FFPE samples, and was performed in three independent assays.

### Human vesicle-associated membrane protein 2 (HuVAMP2) qPCR

To verify the quality of gDNA purified from FFPE tissue and to rule out the presence of PCR inhibitors, patient samples were subjected to a qPCR assay targeting the human VAMP2 gene as described previously [28]. Quantitative standards of pHuVAMP2 was prepared as 10-fold serial dilutions from 10^7^ to 10^0^ copies in the presence of 1 μg *Saccharomyces cerevisiae* transfer RNA (tRNA) carrier nucleic acid per reaction (Sigma-Aldrich). PCR was performed using 10 μl of diluted pHuVAMP2 containing the required plasmid copy numbers or 1 μg of sample gDNA in a final volume of 25 μl. Threshold cycle (Ct) values that were not within the average Ct ± SD (8.9×10^5^ ± 5.4×10^5^ copies/μg) for gDNA from 22Rv1, LNCaP, DU145 and human PBMCs were considered unsuitable for further analysis by qPCR.

### qPCR detection of XMRV and MLV using broad MLV (BMLV) and XMRV specific primers/probe

To detect XMRV and related MLV sequences we used primers (BMLV-F and BMLV-R) and the TaqMan probe (BMLV-Probe) targeting conserved regions in the reverse transcriptase region of XMRV and MoMLV *pol* (Figure 1) (Table 1) [47]. These BMLV primers/probe also have 97-100% nucleotide sequence homology to Friend MLV, Rauscher MLV, Friend spleen focus forming virus, preXMRV-1 and preXMRV-2. In addition, we also used the XMRV specific primers (XMRVIN-F, XMRVIN-R) and probe (XMRVIN-Probe) (Table 1), which target the integrase region of XMRV (Figure 1) as published previously [28]. Quantitative standards of XMRV (pVP62) and MoMLV (pNCS) were prepared by subjecting plasmids to serial dilutions from 10^7^ to 10^0^ copies and 10^6^ to 10^0^ copies, respectively. PCR was performed as published [47] using the required copies of plasmid DNA (in the presence of 1 μg of carrier tRNA) or 1 μg of sample gDNA in a final volume of 25 μl.

### qPCR detection of mouse IAP

qPCR for mouse IAP sequences was used as a marker of mouse DNA contamination as previously described (Table 1) [28]. Serial dilutions (2 fg to 200 ng) of Balb/c gDNA (Sigma) were used as quantitative standards. PCR reactions included the required amounts of standard Balb/c DNA (in the presence of 1 μg of carrier tRNA) or 1 μg of sample gDNA in a final volume of 25 μl.

### Interpretation of qPCR signals detected in patient samples

For detection of XMRV/MLV or mouse DNA contamination, signals that were greater than two standard deviations (SD) from the average Ct of the lowest standard tested (i.e., 1 copy of plasmid or 2 fg of mouse DNA) were considered negative. Samples where a signal was detected within the linear range of the assay and in the majority of independent assays performed, were considered positive.

## Abbreviations

XMRV: Xenotropic murine leukemia virus-related virus XMRV; PC: Prostate cancer; CFS: Chronic fatigue syndrome; gDNA: Genomic DNA; FFPE: Formalin-fixed paraffin-embedded; RNase L: Ribonuclease L; MLV: Murine leukemia virus; qPCR: Quantitative PCR; X-MLV: Xenotropic-MLV; IAP: Intracisternal A-type particle; PBMCs: Peripheral blood mononuclear cells; VAMP2: Human vesicle-associated membrane protein 2; DPS: Deparafinisation solution; ARMS: Amplification refractory mutation system; Ct: Threshold cycle; tRNA: Transfer RNA; SD: Standard deviation; MoMLV: Moloney murine leukemia virus; BMLV: Broad MLV.

## Competing interests

The authors declare that they have no competing interests.

## Authors’ contributions

The project was conceived and funding obtained by GT. Project protocol and ethics was prepared by GT and ACH. Tissue was supplied by JP and JM. Experiments were performed by SDR. Data analysis was performed by SDR and ACH. Statistical analysis was performed by GT. The manuscript was drafted by GT and SDR and all authors were involved in critical revision of the manuscript and approved it for submission.
